# Integrating community services provision for older people living with severe frailty: implications from an England-wide survey

**DOI:** 10.1093/ageing/afaf174

**Published:** 2025-06-30

**Authors:** Richard Green, Sarah Combes, Jenny Harris, Caroline Nicholson

**Affiliations:** University of Surrey - School of Health Sciences, Faculty of Health and Medical Sciences, Kate Granger Building, Priestley Road, Surrey Research Park, Guildford, Surrey GU2 7YH, UK; University of Surrey - School of Health Sciences, Faculty of Health and Medical Sciences, Kate Granger Building, Priestley Road, Surrey Research Park, Guildford, Surrey GU2 7YH, UK; University of Surrey - School of Health Sciences, Faculty of Health and Medical Sciences, Kate Granger Building, Priestley Road, Surrey Research Park, Guildford, Surrey GU2 7YH, UK; University of Surrey - School of Health Sciences, Faculty of Health and Medical Sciences, Kate Granger Building, Priestley Road, Surrey Research Park, Guildford, Surrey GU2 7YH, UK

**Keywords:** frailty, palliative care, community medicine, delivery of healthcare, integrated care, older people

## Abstract

**Background:**

Older people with severe frailty are nearing the end of life and require community services that span the care continuum, integrating older people’s care (e.g. physical function; rehabilitation) and palliative care (e.g. symptoms; what matters most to the person). Little is known about current community service provision to this population.

**Objective:**

To characterise the scope of community service provision to older people with severe frailty in England.

**Methods:**

A cross-sectional online survey design identifying common components and service configurations of community services providing care to older people with severe frailty in England. A self-selecting sample of multidisciplinary care providers from multi-agency community services working with older people with severe frailty with end-of-life care needs.

**Results:**

Two hundred ninety-eight participants from 102 unique services across all English regions reported information on their service, including frailty and end-of-life assessment, service components, training, service costs and service development. Identification and assessment of frailty and end of life were routinely practiced, but approaches to identification varied. Organisations’ training and development were focussed on their own service provision with little focus on inter-organisational and cross-sectoral working.

**Conclusions:**

Community care services responses were varied to this population’s diverse needs, which risks less coordinated practice and poorer care outcomes. Poorly integrated services can only partially meet the needs of older people with severe frailty. Further research is needed to address barriers to integration, including cross-sector collaboration, consistent use of appropriate assessment tools and service innovations driven by older people’s expressed needs.

## Key Points

- Community services must be reconfigured to better meet the needs of older people with severe frailty.
- Service reconfiguration should address barriers in local care infrastructures to enable integration across care settings.
- Barriers include fragmented working, inconsistent assessment tools and lack of patient-oriented service innovation.
- Research is needed to understand needs, outcomes and barriers to integrated care for severe frailty.

## Introduction

Older people with severe frailty have complex needs requiring integrated health and social care, particularly at end of life [1]. A review of service models highlights that both integrated geriatric and palliative care improve quality of life through person-centred care, education and multiprofessional collaboration [2]. However, a care continuum is needed between geriatric care (focused on function and rehabilitation) and palliative care (primarily symptom management) [2]. Effective models incorporate comprehensive assessment, self-management support, decision-making involvement and skilled integrated working [3]. While specialist models offer insights, evidence on who to involve and how to coordinate community services for more integrated, person-centred care remains limited, warranting further exploration of current palliative and frailty care provision [3].

Palliative care supports living well until death, improving both quality and length of life [4], and is most effective when introduced early rather than as terminal care [5]. Severe frailty increases the likelihood of dying within a year [6], yet palliative care for older people living with frailty is often initiated only in the final weeks of life [7]. This delay reflects shifting demographics in where and how older people die. By 2040, palliative care demand in England and Wales will rise by 25%–47% [8], with many frail older people in the community receiving suboptimal end-of-life care [9, 10]. Care home deaths will become most common, requiring a doubling of community end-of-life care capacity [8], making home-based care an international priority [11].

Managing frailty is challenging due to its unpredictable progression and the lack of validated tools to identify those in their last year of life [12]. Clinicians often treat frailty as separate conditions rather than a life-limiting syndrome [13], leading to under-recognition of palliative needs, overestimated prognoses [14] and limited validated tools [14–16]. Consequently, inappropriate treatments persist [17], symptoms go undertreated [18], quality of life declines [19], with poor care planning [20], and frequent hospital transitions occur in the last years of life [19, 21].

Addressing these issues requires integrated, community-based care across health, social and voluntary sectors to meet multidimensional needs (e.g. physical, psychological spiritual) closer to home [22]. This survey examines the current scope and variation in community services for older adults with severe frailty in England, in order to understand how we can better integrate end-of-life care for older adults with severe frailty.

## Methods

### Survey development and content

A 70-item survey (Appendix 1) was developed and pretested by authors R.G. and C.N. Concepts to be measured and items generated were informed by the PALLUP study’s modified e-Delphi study on core palliative care needs in severe frailty [23] and a scoping review on similar needs in older adults with multimorbidity [22]. Consequently, need was conceptualised across physical, psychological, social, spiritual and practical domains [24, 25]. Additional items were drawn from best-practice guidance, including the Gold Standards Framework [26] and National Institute for Health and Care Excellence (NICE) end-of-life care guidelines (NG142) [27].

Desk testing for readability was followed by pretesting via cognitive interviews (*N* = 3) using think-aloud and verbal probing methods [28]. This ensured that questions were consistently meaningful across health, social and voluntary care sectors. In response to feedback, eligibility criteria (Appendix 1, p3) and definitions of key terms (Appendix 2) were added. The final survey covered: (i) identification, assessment and needs of older people with severe frailty; (ii) service structures, components and responses; (iii) workforce training, evidence building and service development; and (iv) participant demographics. Most items were categorical, with some free-text responses.

### Procedures and data collection

The target population included professionals in operational or patient-facing roles within multi-agency, cross-sector community services for older people with severe frailty. An online survey (Qualtrics®) was disseminated via convenience sampling through personal and organisational networks, email and X (formerly Twitter). Email dissemination targeted care organisations across England, identified from online searches and prior contacts [23, 29]. Participants met two inclusion criteria: (i) working in a service supporting older people with severe frailty at home, and (ii) this service had undertaken some form of service improvement for older people in the past 5 years (Appendix 1, p4). Informed consent was obtained, and recruitment ran for seven weeks (October–December 2021).

### Analysis

Frequencies and percentages were calculated using SPSS Statistics 26 software and charts produced in Excel. Missingness patterns for items were explored, and complete cases analysis is presented. Free-text comments were thematically analysed to identify recurring patterns, themes and insights [30].

### Ethics

Favourable ethical opinion was given by the London Brent Ethics Committee (Integrated Research Application System reference number: 277171; study sponsor: University of Surrey).

## Results

### Survey participation

**Figure 1:**
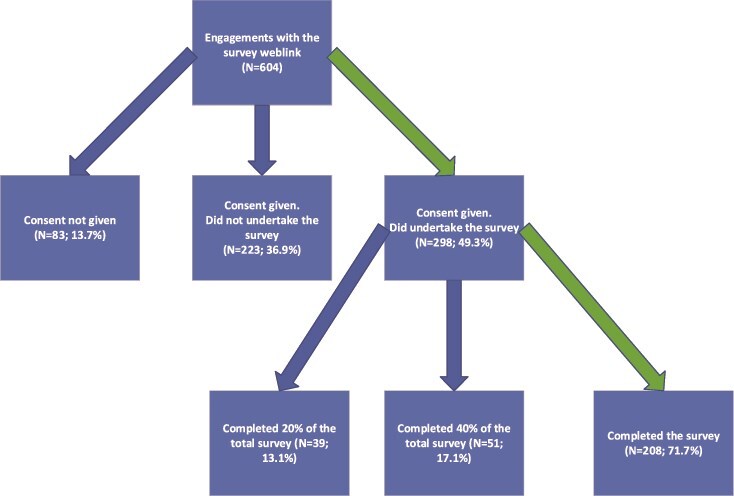
Survey engagement, consent and completion.

In total, 604 people clicked on the survey link (Figure 1) and 298 (49.3%) consented and completed the survey, with the remaining either not providing consent (83, 13.7%) or not proceeding after being reminded of the eligibility criteria (223, 36.9%). The full survey, including demographics and professional background, was completed by 208 people (71.7%), 51 (17.1%) completed up to 40% of the survey and 39 (13.1%) completed up to 20% of the survey. These cut-off points relate to progressing to new pages in the survey. One hundred two unique services were identified by asking participants to name the organisation where they worked, with some participants reporting from the same organisation.

### Characteristics of the sample

Participants were from all English regions, with over a third (34.9%, *N* = 73) from the Southeast. The majority worked in healthcare (82.2%, *N* = 171), with smaller proportions in social, voluntary or multiple sectors (Table 1). Most worked in primary and community services, reflecting the survey’s focus on home-based care, and 80.3% (*N* = 167) were women. The most common self-identified care settings were ‘older people/frailty’ (47.9%, *N* = 99), ‘community nursing’ (27.9%, *N* = 58) and ‘specialist palliative care’ (20.2%, *N* = 42) (see Appendix 3).

**Table 1:** Participant demographics

**Gender of participant**	***N***	**%**
Prefer not to say	9	4.3
Male	32	15.4
Female	167	80.3
**Role type of participant**	***N***	**%**
Service/Patient-facing (clinical/nonclinical)	158	76.0
Organisational leadership or governance	50	24.0
**Region**	***N***	**%**
East Midlands	18	8.7
East of England	16	7.7
Greater London	26	12.5
North East	12	5.8
North West	18	8.7
South East	73	34.9
South West	18	8.7
West Midlands	5	2.4
Yorkshire and the Humber	22	10.6
**Sector(s) of participant’s organisation**	***N***	**%**
Multi-sector care	12	5.8
Voluntary care	7	3.4
Social care	18	8.7
Healthcare	171	82.2
**Setting of participant’s organisation within healthcare sector**	***N***	**%**
Acute	17	9.9
Primary	58	33.9
Secondary	5	2.9
Intermediate	2	1.1
Community	89	52.2

Results are presented across three sections, as per the content of the survey: (i) older people with severe frailty: identification, assessment and needs; (ii) services: structures, components and responses; and (iii) workforce: training, evidence building and service development (further results shown in Appendix 3).

### Survey results

#### Older people with severe frailty: identification, assessment and needs

Frailty identification by participants’ own services was reported by 80.0% (*N* = 236) and end-of-life identification by 92.5% (*N* = 275). Methods varied, with the Clinical Frailty Scale (58.0%, *N* = 93) being the most common, often referred to in free-text responses as the ‘Rockwood Score’. End-of-life identification lacked a dominant method. Beyond clinical tools, clinical discussions were widely used for both frailty (60.9%, *N* = 81) and end-of-life (77.0%, *N* = 117) identification.

The most common assessment method was personalised assessments (47.8%, *N* = 64), such as GP consultations, followed by customised local assessments (30.1%, *N* = 40). Among validated tools, the Comprehensive Geriatric Assessment [31] (41.0%, *N* = 55) and the Integrated Palliative Outcomes Scale [32] (20.9%, *N* = 28) were most frequently reported.

Survey participants also identified which domains of need or capability [22] were addressed by their service (Table 2). Participants identified the needs their services prioritised, with the top three being ‘pain’, ‘long-term disease management’ and ‘mobility’. The most addressed needs were ‘pain’, ‘medicine use’ and ‘recognising existential questions for older people nearing the end of life’. The most unmet needs that participants wished their services would address were ‘rehabilitative care to improve daily living’, ‘loneliness’ and ‘recognising existential questions for older people nearing the end of life’, which were also among the most addressed.

**Table 2:** Domains of needs and capabilities addressed by services (survey question: ‘Which of the following does your service primarily provide support for...? (Please select all that apply)’)

**Domains of need and capabilities**	**Percentage**	**Number**
Physical health needs	94.8	235
Practical daily living needs	69.4	172
Mental health needs	64.1	159
Spiritual, religious and/or cultural needs	43.3	104
Older people’s coping strategies	35.5	88
Older people’s strengths	33.1	82

*n =* 248—participants could select multiple options.

Chi-square tests were conducted to examine variations in needs assessment by type of need across two newly combined variables: service type (acute/secondary, primary, community/intermediate care) and sector (healthcare, nonhealthcare). For service type, a significant difference was observed in addressing mental health needs (χ^2^(2) = 12.61, *P* = .002), with community/intermediate care less likely to address them compared to acute/secondary and primary care. Descriptive analysis showed that community/intermediate care services (51.6%) were less likely to report addressing mental health needs than primary (79.3%) and acute/secondary services (72.7%). For sector, significant variations were found in addressing spiritual/religious/cultural needs [χ^2^(1) = 10.708, *P* = .001] and older people’s strengths [χ^2^(1) = 6.469, *P* = .011]. Healthcare services were less likely to provide support for spiritual/religious/cultural needs (37.6%) compared to services in the social, voluntary or other sectors (72.0%). Healthcare services were also less likely to provide support for older people’s strengths (30.4%) compared to services in the social/voluntary sectors (56.0%). Chi-square results are shown in Appendix 4.

#### Services: structures, components and responses

**Figure 2:**
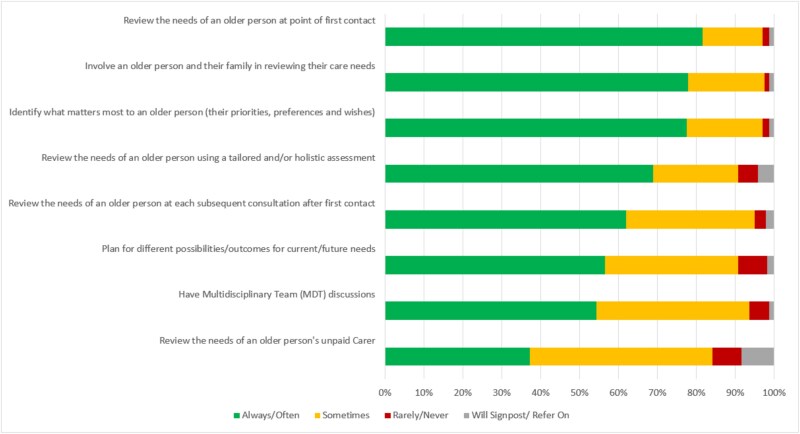
Service components for patient assessment (survey question: ‘Thinking about the care that is provided in your service, how often do the following take place...?’).

**Figure 3:**
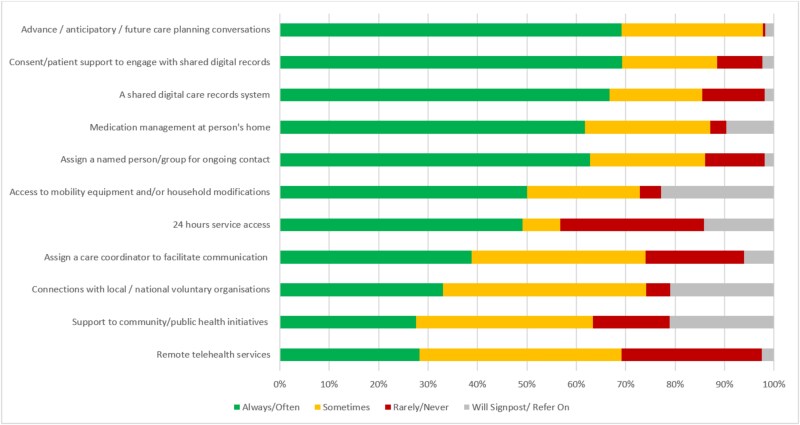
Service responses for addressing patient need (survey question: ‘Which of the following service structures does your service provide?’).

Service structures, components and responses to address identified needs were reported across the survey items (Figures 2 and 3).

The least commonly reported service components for assessing older people’s needs included reviewing unpaid carers’ needs, using tailored/holistic tools and planning for current and future needs with older people (Figure 2). The least common service structures and responses were 24-hour access, remote telehealth services (e.g. video consultations) and support for community/public health initiatives promoting health, wellbeing and quality of life. The most frequently rated ‘Will Signpost/Refer On’ services included access to mobility equipment, connections with voluntary organisations and support for public health initiatives (Figure 3).

#### Workforce: training, evidence building and service development

Participants were asked about training, evidence building and service development in their service. For the question, ‘Does your service/organisation provide education and training for...? (Please select all that apply)’, the most common staff training offered was safeguarding vulnerable people (93.7%) and end-of-life care for older people with severe frailty (89.4%). The least common were training for outreach to unpaid carers (45.5%), outreach to other care sectors (53.1%) and support for other services in end-of-life care (63.3%) (see Appendix 3).

Responses to the free-text question: ‘How has your service changed in response to the COVID-19 pandemic?’ (*N* = 116) were thematically analysed by RG and then reviewed with CN. Findings showed that most services responded to the COVID-19 pandemic by (i) increasing remote/telephone consultations (*N* = 41) (e.g. ‘More telephone triage’[ID089] and ‘Heavier reliance on telephone support which has impacted on access for people with hearing impairment’[ID158]), (ii) adapting care pathways due to reduced service capacity (*N* = 24) (e.g. ‘changed pathways of care with a focus on more acute presentation and hospital avoidance’[ID112] and ‘reduced day hospice services’[ID037]), (iii) modifying working practices and roles (*N* = 22) (e.g. ‘Looking at new roles within the service to support the local population such as palliative support, diabetic support’[ID64]) and (iv) adopting more collaborative working practices (*N* = 6) (e.g. ‘Joint clinic with community health trust now has formal MDT weekly’[ID141]).

## Discussion

This paper explores service structures and practices for the provision of care to older people living with severe frailty in the community in England. The results evidence ways in which existing structures and practices could be improved to better meet the needs of this population.

While survey participants reported high levels of engagement in identifying severe frailty and end-of-life care needs, there was no consistent approach to assessment. Services often relied on locally developed tools and clinical judgement to determine older people’s needs. This lack of standardisation is concerning, as clinical assessment and discussion—while essential—may not always capture the full range of older people’s expressed needs. If these remain the dominant methods of assessment, an important question for older people’s care globally is how to ensure they are used in evidence-based ways that reflect older people’s priorities and lived experiences.

Results also indicate that services prioritise safeguarding training and organisation-specific care approaches over cross-sector training and education. This suggests a limited emphasis on collaborative working and a missed opportunity to develop shared competencies across health, social and voluntary care sector providers. The absence of such training likely reinforces the fragmented nature of service provision and limits the extent to which services can be effectively integrated.

Service developments reported in this survey appear to have been primarily driven by system demands during the COVID-19 pandemic rather than by older people’s expressed needs. The timing of the survey, alongside the prominence of COVID-19-related themes in free-text responses, suggests that most innovations—such as the expansion of remote consultations—were reactive rather than proactively designed to improve patient experience. While remote service delivery may offer staff efficiency and infection control benefits, it also risks exacerbating inequities in access for older people with severe frailty, particularly those facing digital exclusion [22]. The low volume of reported service changes focused on collaboration or explicitly responding to patient-reported needs suggests that service adaptation was largely system-led rather than patient-driven. The survey responses demonstrate that service provision remains heavily weighted towards addressing physical needs, particularly pain management, mobility and medication use. While these are critical aspects of care, nonphysical needs—such as loneliness, existential concerns and reablement—were reported as needs that care providers desired to meet but not considered as in their remit to address. Chi-Square analysis added to this understanding, showing that nonhealthcare providers are more likely to respond to spiritual and cultural needs.

By service type, acute and secondary care and primary care were more likely to address mental health needs than community and intermediate services. This finding may indicate a gap in mental health need identification and assessment in the community, where specific mental health needs for this population (e.g. arising from loneliness/bereavement) may not be assessed, being seen as a ‘natural’ part of ageing [23]. These findings align with previous research (from care providers’ perspectives) indicating that older people’s expressed needs often extend beyond physical health [22], yet health services continue to prioritise medical and functional concerns. Addressing these broader needs requires a more integrated and continuous approach, one that acknowledges how complex needs are dynamic and evolve over time, requiring multidisciplinary and multi-sectoral services that can shape to differing needs.

Beyond these broad multidimensional needs is the evident tendency to focus on deficits rather than strengths. Table 1 showed how services were predominantly prioritising needs rather than supporting older people’s coping strategies, resilience and retained capabilities. While this reflects existing constraints on care delivery, it challenges efforts to implement genuinely personalised care that aligns with older people’s values and priorities. Chi-square analysis indicated a weak but significant relationship for nonhealthcare providers being more likely to provide support for older people’s strengths, suggesting that some nonhealthcare providers may have skills and experience in this area that other providers could benefit from. In the UK, there is growing recognition of the need to shift towards nonpharmacological interventions to manage multimorbidity and complexity in ageing populations, including those with severe frailty [33]. However, achieving this in practice requires structural changes in service delivery to better support a more holistic, person-centred approach.

Encouragingly, the needs prioritised by services in this survey are broadly consistent with those outlined in recent frameworks for integrated geriatric and palliative care [2, 3]. However, the challenge now is how services globally can be reconfigured within the context of locally funded infrastructures to meet the needs of older people living with severe frailty, rather than being oriented around what services can provide.

### Strengths and limitations

This cross-sectoral survey examines how care services support older people with severe frailty in England. Due to the absence of a national data frame for cross-sector providers, convenience sampling was necessary. Estimating the true number of community service providers is difficult, but from registered providers of adult social care alone, the number is likely to be in the tens of thousands. However, with little collected data on the types and components of care provision available for older people with severe frailty in England, this survey provides a snapshot of current practice across sectors.

The sample was predominantly healthcare professionals, particularly from community, older people’s and palliative care backgrounds. Social and voluntary sector providers were underrepresented despite efforts to ensure accessibility across sectors, possibly due to the medicalised language of the survey and the mode of delivery in terms of who has time and access to emails in their daily work. This highlights challenges in cross-sector engagement and the need for inclusive language and alternative modes of delivery in future research. The survey was also conducted during the COVID-19 pandemic, which may also have affected participation and response patterns.

As a self-selecting, nonrepresentative survey, sophisticated statistical analyses were not appropriate, and reporting variation was challenging. Sample characteristics were highly skewed (e.g. by gender, sector and service type), limiting comparisons between groups. Even where representation was more balanced (e.g. by region), comparisons were problematic as participants could select multiple responses, making subgroup analysis unreliable. These limitations offer methodological insights for future quantitative studies capturing cross-sector perspectives on frailty care.

### Implications for policy, practice and research

These results provide a snapshot of how community services support older people with severe frailty. Despite recent legislative changes in England, including statutory palliative care requirements within Integrated Care Boards (ICBs) [34], traditional fragmented care models persist. Service reconfiguration must address barriers to integration within the evolving Integrated Care Systems infrastructure. Our findings highlight challenges in care coordination, cross-sector collaboration and engagement beyond organisational boundaries, aligning with a recent report on the *Ambitions Framework*, which found community involvement to be the least addressed of the six palliative care priorities [35]. Research by Marie Curie found that only 35% of ICBs report that they significantly or fully understand palliative and end-of-life care population health needs [36]. These challenges are receiving increasing attention internationally, such as through the European Association of Palliative Care’s Reference Group on Aging and Palliative Care [37]. Current palliative care policy and practice do not fully accommodate the needs of older people living and dying with frailty [38]. This survey reinforces the need for service redesign that places older people’s holistic needs at the forefront, rather than focusing predominantly on physical symptoms. Encouragingly, the findings align with emerging service frameworks for integrated older people’s care [2, 3], which are also informing palliative and integrated care policies internationally [39–41]. However, further research is needed to optimise implementation within and across organisations.

Our findings suggest existing service configurations are not yet prepared for the integration. Barriers include:

- Lack of cross-sector collaborative working
- Inconsistent use of tailored assessment tools
- Limited service development driven by older people’s expressed needs

The components and responses of existing services in this survey appear well-matched to these service frameworks is encouraging, yet further research is necessary to understand how to make the best use of these through collaborative and integrated systems both within and across organisations. Further, service developments reported in this survey indicate that existing service configurations are not well prepared for the desired integration expressed in newly emerging policies. Barriers to greater integration identified in this survey include a lack of assessment tools tailored to this population and consistency of tool use and the current lack of service development being driven by older people’s expressed needs. These challenges mirror those identified in a recent collaborative development of a service framework for older people living and dying with severe frailty [42], which highlights the following sector-wide issues:

- Limited involvement of older people in service design
- Poor cross-sector collaboration
- Absence of a common language
- Workforce instability
- Insufficient funding for cross-sector initiatives

Further research is required to better understand the needs, experiences and most appropriate outcomes for older people with severe frailty and to identify effective strategies for fostering integrated, person-centred care.

## Conclusion

This survey highlights critical gaps in the provision of community care for older people with severe frailty in England, particularly in addressing holistic needs, integrating care across sectors and ensuring service development is led by older people’s expressed needs rather than system constraints. The findings underscore the need for greater consistency in assessment approaches, improved training in cross-sector collaboration and cultural shifts in care provision that move beyond a deficit-focused model to one that values older people’s strengths and preferences.

To achieve meaningful service improvements, recent policy shifts towards integrated care must be accompanied by changes in working cultures, shared language and funding mechanisms that support true cross-sector collaboration. Further research is needed to explore how to overcome these barriers and design services that more effectively meet the complex and dynamic needs of older people living with severe frailty.

## Supplementary Material

aa_24_1743_File002_afaf174

aa_24_1743_File003_afaf174

aa_24_1743_File004_afaf174

aa_24_1743_File005_afaf174
