# Supplementary material for: Integrating community services provision for older people living with severe frailty: implications from an England-wide survey
Source: Age Ageing. 2025 Jun 30;54(6):afaf174. doi: 10.1093/ageing/afaf174 (PMC12207214; doi:10.1093/ageing/afaf174)
Supplement: aa_24_1743_File003_afaf174 [file aa_24_1743_file003_afaf174.docx]

**Appendix 2 – Survey Participant Criteria and Definitions of Key Terms**

**Introduction**
 
This **England-wide** online survey will explore service responses and improvements from care services to meet the needs of older people living at home with advancing frailty. Findings will support co-produced resources to improve the support provided both to older people living at home and their carers; to enable the best possible quality of life in their final years.

This survey is aimed at services providing care in the community for older people living with advancing frailty in and across Health care, Social care, and Voluntary care (including community groups and/or social enterprise, not-for-profit, and charity organisations).

This survey is for those who are working in an operational role and those who are patient/client facing.

You may hold several roles within your service. If so, please select a single perspective to answer this survey consistently from.

If you can think of anyone else providing services to older people with advancing frailty who may be interested in completing this survey we would be very grateful if you would forward the survey details on to them.

The questions in the survey are informed by care needs identified in our previous research.**Please read our definitions below.**

**Definitions**

**Frailty** is a gradual loss of in-built reserves, leaving people vulnerable to dramatic, sudden changes in health triggered by seemingly small events such as a minor infection or a change in medication or environment.

**Older people living with severe frailty** are those who require help with all outside activities and a high level of personal care support when at home. They have complex care needs and are thought to be in the final years of life.

**Home** is defined as a domestic dwelling where someone resides, including residential care settings (e.g. residential care homes, sheltered housing/assisted living, and retirement villages). In our study it does not include care homes with on-site nursing.

**Palliative care need** is understood as having a requirement for comfort and support with a condition that is life limiting, that can be met by health, social, or voluntary care. This is often necessary in the **final years of life**, which covers a period that is difficult to estimate and may be months or years dependent on a person's needs.

**A service** is a system designed to address care needs and can be provided by a single organisation or can be across multiple organisations.

**Your organisation** is the named place/group where you work

**Your role** is what you do in your organisation (if you occupy multiple roles, please select one role to answer the survey from a single perspective).
