# Supplementary material for: Integrating community services provision for older people living with severe frailty: implications from an England-wide survey
Source: Age Ageing. 2025 Jun 30;54(6):afaf174. doi: 10.1093/ageing/afaf174 (PMC12207214; doi:10.1093/ageing/afaf174)
Supplement: aa_24_1743_File004_afaf174 [file aa_24_1743_file004_afaf174.docx]

**Appendix 3 – Supplementary Data**

**Section 1**

**Table A: Do you identify frailty in your service?**

| **Response** | **Number (N)** | **Percentage (%)** |
| --- | --- | --- |
| Don't Know / Unsure | 8 | 4.9 |
| No | 22 | 13.5 |
| Yes | 133 | 81.6 |
| Total | 163 | 100 |

**Table B: How is frailty identified? (Please select all that apply)**

| **Method of Frailty Identification** | **Number (N)** | **Percentage (%)** |
| --- | --- | --- |
| Clinical discussion(s) with the Multidisciplinary Team | 77 | 57.9 |
| Clinical discussion(s) with the Multidisciplinary Team and Family / Older Person | 81 | 60.9 |
| Timed-up-and-go test (<12s) | 19 | 14.3 |
| Grip strength | 6 | 4.5 |
| PRISMA 7 questionnaire | 5 | 3.8 |
| Clinical frailty scale (CFS) | 71 | 53.4 |
| Edmonton frail scale | 3 | 2.3 |
| Barthel Scale/Index (BI) | 13 | 9.8 |
| Population-level tool e.g. the electronic Frailty Index (eFI) | 32 | 19.6 |

**Table C: Do you identify frailty in your service?**

| **Response** | **Number (N)** | **Percentage (%)** |
| --- | --- | --- |
| Don't Know / Unsure | 2 | 1.2 |
| No | 9 | 5.5 |
| Yes | 152 | 93.3 |
| Total | 163 | 100 |

**Table D: How are those thought to be nearing the end of life identified? (Please select all that apply)**

| **Identification Method** | **Number (N)** | **Percentage (%)** |
| --- | --- | --- |
| Clinical discussion(s) with the Multidisciplinary Team | 95 | 62.5 |
| Clinical discussion(s) with the MDT and Family / Older Person | 117 | 77.0 |
| The Gold Standards Framework Proactive Identification Guidance (PIG) | 77 | 50.7 |
| The Surprise Question | 71 | 46.7 |
| The Supportive and Palliative Care Indicators Tool [SPICT] | 30 | 19.7 |
| Planned end of life meeting(s) | 52 | 34.2 |
| Identifying awareness of increasing end of life care need | 92 | 60.9 |

**Table E: Which of the following tailored and/or holistic assessment methods does your service use? (Please select all that apply)**

| **Assessment Method** | **Number (N)** | **Percentage (%)** |
| --- | --- | --- |
| Palliative Outcome Scale (POS) | 5 | 3.7 |
| Integrated Palliative Outcome Scale (IPOS) | 28 | 20.9 |
| Specialist Palliative Care Assessment (e.g. OACC suite) | 23 | 17.2 |
| Comprehensive Geriatric Assessment (CGA) | 55 | 41.0 |
| Service-specific assessment template | 40 | 30.1 |
| Personalised assessment (e.g. GP consultation) | 64 | 47.8 |
| Patient or carer-held symptom monitoring tool | 10 | 7.5 |
| Goals of care to structure consultations | 25 | 18.7 |

**Table F: Which of the following methods does your service use to support unpaid carers?**

| **Unpaid Carer Support Method** | **Number (N)** | **Percentage (%)** |
| --- | --- | --- |
| End of life focus care tools (e.g. CSNAT) | 12 | 10.1% |
| A local council carers assessment | 57 | 47.9% |
| An assessment template specific to your service | 11 | 9.2% |
| Other (please describe) | 27 | 22.9% |

**The following questions on needs were branched questions only answered by those who selected the relevant need options as shown in Table 2 of the article.**

**Table G: Which of the following physical health needs does your service primarily provide support for...? (N=144)**

| **Physical Health Need** | **Always / Often** | **Sometimes** | **Rarely / Never** | **Will Signpost / Refer On** | **N/A for Service** | **Don't Know / Unsure** |
| --- | --- | --- | --- | --- | --- | --- |
| Pain | 116 (80.6%) | 19 (13.2%) | 1 (0.7%) | 7 (4.9%) | 1 (0.7%) | – |
| Mobility | 101 (70.1%) | 24 (16.7%) | 2 (1.4%) | 16 (11.1%) | 1 (0.7%) | – |
| Hearing/ Visual | 38 (26.4%) | 50 (34.7%) | 12 (8.3%) | 35 (24.3%) | 5 (3.5%) | 3 (2.1%) |
| Sleep Disturbance | 44 (30.6%) | 60 (41.7%) | 15 (10.4%) | 19 (13.2%) | 4 (2.8%) | 1 (0.7%) |
| Medicines Use | 93 (64.6%) | 31 (21.5%) | 2 (1.4%) | 15 (10.4%) | 2 (1.4%) | 1 (0.7%) |
| Long-Term Conditions | 97 (67.4%) | 32 (22.2%) | 3 (2.1%) | 9 (6.3%) | 3 (2.1%) | – |
| Memory Loss | 59 (41.0%) | 42 (29.2%) | 9 (6.3%) | 30 (20.8%) | 4 (2.8%) | – |
| Acute Confusion | 74 (51.4%) | 37 (25.7%) | 8 (5.6%) | 20 (13.9%) | 5 (3.5%) | – |

**Table H: Which of the following psychological / mental health needs does your service primarily provide support for...? (N=99)**

| **Psychological / Mental Health Need** | **Always / Often** | **Sometimes** | **Rarely/Never** | **Will Signpost / Refer On** | **N/A for Service** | **Don't Know / Unsure** |
| --- | --- | --- | --- | --- | --- | --- |
| Low Mood | 62 (62.6%) | 28 (28.3%) | – | 7 (7.1%) | – | – |
| Anxiety | 60 (60.6%) | 28 (28.3%) | 1 (1.0%) | 8 (8.1%) | – | – |
| Managing Loss / Bereavement | 41 (41.4%) | 27 (27.3%) | 5 (5.1%) | 24 (24.2%) | – | – |
| Loneliness | 32 (32.3%) | 31 (31.3%) | 7 (7.1%) | 25 (25.3%) | 1 (1.0%) | 1 (1.0%) |

**Table I: Which of the following practical needs does your service primarily provide support for...? (N=108)**

| **Practical Need** | **Always / Often** | **Sometimes** | **Rarely / Never** | **Will Signpost / Refer On** | **N/A for Service** | **Don't Know / Unsure** |
| --- | --- | --- | --- | --- | --- | --- |
| Staying Active | 38 (35.2%) | 39 (36.1%) | 11 (10.2%) | 17 (15.7%) | 2 (1.9%) | 1 (0.9%) |
| Rehabilitative Care (Daily Living Abilities) | 34 (31.5%) | 37 (34.3%) | 8 (7.4%) | 23 (21.3%) | 4 (3.7%) | 2 (1.9%) |
| Staying Connected with People and Places | 24 (22.2%) | 37 (34.3%) | 17 (15.7%) | 23 (21.3%) | 2 (1.9%) | 5 (4.6%) |
| Relationships with Family | 41 (38.0%) | 38 (35.2%) | 10 (9.3%) | 15 (13.9%) | 1 (0.9%) | 3 (2.8%) |

**Table J: Which of the following spiritual, religious, and or cultural needs does your service primarily provide support for...? (N=61)**

| **Spiritual, Religious, and or Cultural Need** | **Always / Often** | **Sometimes** | **Rarely / Never** | **Will Signpost / Refer On** | **Don't Know / Unsure** |
| --- | --- | --- | --- | --- | --- |
| Spiritual, Religious, and/or Cultural Beliefs and Practices | 29 (47.5%) | 20 (32.8%) | 2 (3.3%) | 9 (14.8%) | 1 (1.6%) |
| Culturally Sensitive Care (e.g., interpreters, diet) | 37 (60.7%) | 13 (21.3%) | 4 (6.6%) | 7 (11.5%) | – |
| Existential Questions in Final Years of Life | 41 (67.2%) | 15 (24.6%) | 3 (4.9%) | 2 (3.3%) | – |

**Section 2**

**Table K: How does your organisation provide 24 hours service access?**

| **Service Access Method** | **Number (N)** | **Percentage (%)** |
| --- | --- | --- |
| Having an organisational single point of contact that is always available | 45 | 72.6 |
| Providing some cover and then signposting to other services at other times | 13 | 21.0 |

**Table L: How does your service provide medications management at home? (Please select all that apply)**

| **Medications Management Method** | **Number (N)** | **Percentage (%)** |
| --- | --- | --- |
| Specialist palliative care medications | 72 | 60.0 |
| Arranging just-in-case medications in home care settings | 93 | 77.5 |
| Working with community pharmacists | 82 | 68.3 |
| Review of medicines use | 101 | 84.2 |

**Table M: Which technology-facilitated communication methods does your service use for the facilitation and delivery of care remotely? (Please select all that apply)**

| **Remote Care Communication Method** | **Number (N)** | **Percentage (%)** |
| --- | --- | --- |
| Telephone | 84 | 95.5 |
| Video Calls | 78 | 88.6 |
| Text Services | 43 | 48.9 |
| Email | 51 | 58.0 |
| Remote monitoring technologies for health conditions | 20 | 22.7 |
| Smartphone Apps | 14 | 15.9 |

**Table N: How does your service provide advance / anticipatory / future care planning? (Please select all that apply)**

| **Method of advance / anticipatory / future care planning** | **Number (N)** | **Percentage (%)** |
| --- | --- | --- |
| Conversations over time | 117 | 96.7 |
| Conversations with others (e.g. surrogate decision-makers) | 84 | 69.4 |
| Providing information about Power of Attorney | 78 | 64.5 |
| Support with planning for future care decisions and preferences | 102 | 84.3 |
| Support with managing practical affairs associated with dying | 37 | 30.8 |
| Consent and information regarding shared Digital Records | 83 | 68.6 |

**Section 3**

**Table O: Does your service / organisation provide education and training for...? (Please select all that apply)**

| **Education or Training Method** | **Yes** | **No** | **Don't Know** | **N/A for my service** | **Total** |
| --- | --- | --- | --- | --- | --- |
| Your staff to promote confidence and capability in end-of-life care for older people with advancing frailty | 127 (90.1%) | 15 (10.6%) | 3 (2.1%) | 2 (1.4%) | 141 |
| Other services to promote confidence and capability in end-of-life care for older people with advancing frailty | 76 (55.9%) | 44 (32.4%) | 21 (15.4%) | 6 (4.4%) | 136 |
| Communication that considers any sensory needs an older person may have (e.g. hearing or sight difficulties) | 82 (60.3%) | 46 (33.8%) | 17 (12.5%) | 2 (1.5%) | 136 |
| Communication with other care providers effectively to manage transitions in care | 90 (66.2%) | 36 (26.5%) | 17 (12.5%) | 4 (2.9%) | 136 |
| On safeguarding for vulnerable people | 134 (93.7%) | 9 (6.3%) | 1 (0.7%) | 1 (0.7%) | 143 |
| Outreach to support/educate unpaid carers of older people | 50 (34.7%) | 60 (41.7%) | 21 (14.6%) | 15 (10.4%) | 144 |
| Outreach to other care sectors | 60 (48%) | 53 (42.4%) | 24 (19.2%) | 10 (8%) | 125 |
| Support services for the wellbeing of staff/volunteers | 90 (66.2%) | 36 (26.5%) | 14 (10.3%) | 7 (5.1%) | 136 |
| Appropriate information and education available for patients and families in accessible formats | 97 (67.4%) | 26 (18.1%) | 16 (11.1%) | 7 (4.9%) | 144 |

**Table P: Which professions or specialist groups will you signpost/refer people to...? (Please select all that apply)**

| **Specialist Group** | **Always/Often (%)** | **Sometimes (%)** | **Rarely/Never (%)** | **Unavailable (%)** | **N/A for my service (%)** | **Total Responses (%)** |
| --- | --- | --- | --- | --- | --- | --- |
| Older People / Frailty | 43 (28.7%) | 67 (44.7%) | 11 (7.3%) | 7 (4.7%) | 15 (10%) | 150 (100%) |
| Community Nurse | 91 (60.7%) | 45 (30%) | 4 (2.7%) | 0 (0%) | 8 (5.3%) | 150 (100%) |
| Mental Health | 46 (30.7%) | 91 (60.7%) | 9 (6%) | 2 (1.3%) | 1 (0.7%) | 150 (100%) |
| Direct Social Care (e.g. Domiciliary Care) | 79 (52.7%) | 54 (36%) | 4 (2.7%) | 3 (2%) | 9 (6%) | 150 (100%) |
| Occupational Therapy | 83 (55.3%) | 56 (37.3%) | 4 (2.7%) | 0 (0%) | 7 (4.7%) | 150 (100%) |
| Physiotherapy | 81 (54%) | 60 (40%) | 3 (2%) | 1 (0.7%) | 4 (2.7%) | 150 (100%) |
| Dietician | 45 (30%) | 79 (52.7%) | 17 (11.3%) | 4 (2.7%) | 3 (2%) | 150 (100%) |
| Community Pharmacy | 41 (27.3%) | 77 (51.3%) | 26 (17.3%) | 0 (0%) | 3 (2%) | 150 (100%) |
| Paramedic | 19 (12.7%) | 56 (37.3%) | 55 (36.7%) | 1 (0.7%) | 14 (9.3%) | 150 (100%) |
| Voluntary Worker | 27 (18%) | 85 (56.7%) | 24 (16%) | 4 (2.7%) | 6 (4%) | 150 (100%) |
| Faith Worker | 15 (10%) | 44 (29.3%) | 68 (45.3%) | 9 (6%) | 8 (5.3%) | 150 (100%) |
| Social Worker | 69 (46%) | 65 (43.3%) | 7 (4.7%) | 3 (2%) | 3 (2%) | 150 (100%) |
| Chiropodist / Podiatrist | 29 (19.3%) | 79 (52.7%) | 29 (19.3%) | 3 (2%) | 6 (4.7%) | 150 (100%) |
| Specialist Palliative Care | 66 (44%) | 55 (36.7%) | 14 (9.3%) | 2 (1.3%) | 9 (6%) | 150 (100%) |
| Social Prescriber | 34 (22.7%) | 65 (43.3%) | 34 (22.7%) | 5 (3.3%) | 6 (4%) | 150 (100%) |
| Speech Therapist | 28 (17.3%) | 76 (50.7%) | 37 (24.7%) | 2 (1.3%) | 3 (2%) | 150 (100%) |
| Dental Care | 11 (7.3%) | 39 (26%) | 76 (50.7%) | 9 (6%) | 7 (4.7%) | 150 (100%) |

**Table Q: Does your service engage with other providers to identify those who might benefit from your service?**

| **Response** | **Number (N)** | **Percentage (%)** |
| --- | --- | --- |
| No | 30 | 20 |
| Yes | 120 | 80 |
| Total | 150 | 100 |

**Table R: Does your service have systems that support collecting data and estimating service costs?**

| **Response** | **Number (N)** | **Percentage (%)** |
| --- | --- | --- |
| Don't Know / Unsure | 41 | 27.9 |
| No | 15 | 10.2 |
| Yes | 91 | 61.9 |
| Total | 147 | 100.0 |

**Table S: Has your service undertaken any evidence building (e.g. reviews, evaluations) about your service within the last five years?**

| **Response** | **Number (N)** | **Percentage (%)** |
| --- | --- | --- |
| Don't Know / Unsure | 52 | 35.4 |
| No | 19 | 12.9 |
| Yes | 76 | 51.7 |
| Total | 147 | 100 |
