# Supplementary material for: Integrating community services provision for older people living with severe frailty: implications from an England-wide survey
Source: Age Ageing. 2025 Jun 30;54(6):afaf174. doi: 10.1093/ageing/afaf174 (PMC12207214; doi:10.1093/ageing/afaf174)
Supplement: aa_24_1743_File005_afaf174 [file aa_24_1743_file005_afaf174.docx]

**Appendix 4**

**Supplementary Table 1: Chi-Square Analysis of Needs Assessment by Service Type (Acute and Secondary – Primary – Community and Intermediate)**

| **Need Type** | **Service Type** | **Count** | **% within Service Type** | **% of Total** | **Pearson Chi-Square** | **df** | **p-value** |
| --- | --- | --- | --- | --- | --- | --- | --- |
| **Physical Health Needs** | Acute and Secondary | 21 | 95.5% | 12.3% | 0.573 | 2 | 0.751 |
|  | Primary | 57 | 98.3% | 33.3% |  |  |  |
|  | Community and Intermediate | 89 | 97.8% | 52.0% |  |  |  |
|  | Total | 167 | 97.7% | 97.7% |  |  |  |
| **Mental Health Needs** | Acute and Secondary | 16 | 72.7% | 9.4% | 12.610 | 2 | **0.002** |
|  | Primary | 46 | 79.3% | 26.9% |  |  |  |
|  | Community and Intermediate | 47 | 51.6% | 27.5% |  |  |  |
|  | Total | 109 | 63.7% | 63.7% |  |  |  |
| **Practical Daily Living Needs** | Acute and Secondary | 16 | 72.7% | 9.4% | 4.459 | 2 | 0.108 |
|  | Primary | 34 | 58.6% | 19.9% |  |  |  |
|  | Community and Intermediate | 68 | 74.7% | 39.8% |  |  |  |
|  | Total | 118 | 69.0% | 69.0% |  |  |  |
| **Spiritual, Religious, and/or Cultural Needs** | Acute and Secondary | 12 | 54.5% | 7.0% | 4.448 | 2 | 0.108 |
|  | Primary | 17 | 29.3% | 9.9% |  |  |  |
|  | Community and Intermediate | 32 | 35.2% | 18.7% |  |  |  |
|  | Total | 61 | 35.7% | 35.7% |  |  |  |
| **Older People's Strengths** | Acute and Secondary | 7 | 31.8% | 4.1% | 0.090 | 2 | 0.956 |
|  | Primary | 17 | 29.3% | 9.9% |  |  |  |
|  | Community and Intermediate | 26 | 28.6% | 15.2% |  |  |  |
|  | Total | 50 | 29.2% | 29.2% |  |  |  |

**Notes:** Pearson Chi-Square tests were used to examine differences in needs assessment across service types. Significant results (p < 0.05) are highlighted in bold.
Data is drawn from responses to the following questions:
Which of the following does your service primarily provide support for...? (Please select all that apply)
Within which setting is your primary role?

**Supplementary Table 2: Chi-Square Analysis of Needs Assessment by Sector (Healthcare vs. Non-Healthcare)**

| **Need Type** | **Sector** | **Number (N)** | **% within Sector** | **% of Total** | **Pearson Chi-Square** | **df** | **p-value** |
| --- | --- | --- | --- | --- | --- | --- | --- |
| **Physical health needs** | Healthcare | 176 | 97.2% | 85.4% | 1.836 | 1 | 0.175 |
|  | Non-healthcare | 23 | 92.0% | 11.2% |  |  |  |
|  | Total | 199 | 96.6% | 96.6% |  |  |  |
| **Mental health needs** | Healthcare | 118 | 65.2% | 57.3% | 0.454 | 1 | 0.501 |
|  | Non-healthcare | 18 | 72.0% | 8.7% |  |  |  |
|  | Total | 136 | 66.0% | 66.0% |  |  |  |
| **Practical daily living needs** | Healthcare | 125 | 69.1% | 60.7% | 1.261 | 1 | 0.261 |
|  | Non-healthcare | 20 | 80.0% | 9.7% |  |  |  |
|  | Total | 145 | 70.4% | 70.4% |  |  |  |
| **Spiritual, religious, and/or cultural needs** | Healthcare | 68 | 37.6% | 33.0% | 10.708 | 1 | **0.001** |
|  | Non-healthcare | 18 | 72.0% | 8.7% |  |  |  |
|  | Total | 86 | 41.7% | 41.7% |  |  |  |
| **Older people’s strengths** | Healthcare | 55 | 30.4% | 26.7% | 6.469 | 1 | **0.011** |
|  | Non-healthcare | 14 | 56.0% | 6.8% |  |  |  |
|  | Total | 69 | 33.5% | 33.5% |  |  |  |

**Notes:** Pearson Chi-Square tests were used to examine differences in needs assessment across service types. Significant results (p < 0.05) are highlighted in bold.
Data is drawn from responses to the following questions:
Which of the following does your service primarily provide support for...? (Please select all that apply)
Within which sector is your primary role?
