# Supplementary material for: Integrating community services provision for older people living with severe frailty: implications from an England-wide survey
Source: Age Ageing. 2025 Jun 30;54(6):afaf174. doi: 10.1093/ageing/afaf174 (PMC12207214; doi:10.1093/ageing/afaf174)
Supplement: Supplementary file 4 [file aa_24_1743_file002_afaf174.docx]

PALLUP Phase 2 Survey

Start of Block: Information and Consent

**The PALLUP Study** 

 

**Service responses and improvements in community care provision to older people living with advancing frailty in the final years of life**

**Introduction**

 

This **England-wide** online survey will explore service responses and improvements from care services to meet the needs of older people living at home with advancing frailty. Findings will support co-produced resources to improve the support provided both to older people living at home and their carers; to enable the best possible quality of life in their final years. 

 

This survey is aimed at services providing care in the community for older people living with advancing frailty in and across Health care, Social care, and Voluntary care (including community groups and/or social enterprise, not-for-profit, and charity organisations).

This survey is for those who are working in an operational role and those who are patient/client facing.   	
 You may hold several roles within your service. If so, please select a single perspective to answer this survey consistently from.

If you can think of anyone else providing services to older people with advancing frailty who may be interested in completing this survey we would be very grateful if you would forward the survey details on to them.

 

The questions in the survey are informed by care needs identified in our previous research. **Please read our definitions below.**

 

**Definitions**

 

**Frailty** is a gradual loss of in-built reserves, leaving people vulnerable to dramatic, sudden changes in health triggered by seemingly small events such as a minor infection or a change in medication or environment. 

 

**Older people living with advancing frailty** are those who require help with all outside activities and a high level of personal care support when at home. They have complex care needs and are thought to be in the final years of life.

 

**Home** is defined as a domestic dwelling where someone resides, including residential care settings (e.g. residential care homes, sheltered housing/assisted living, and retirement villages). In our study it does not include care homes with on-site nursing.
**Palliative care need** is understood as having a requirement for comfort and support with a condition that is life limiting, that can be met by health, social, or voluntary care. This is often necessary in the **final years of life**, which covers a period that is difficult to estimate and may be months or years dependent on a person's needs.
**A service** is a system designed to address care needs and can be provided by a single organisation or can be across multiple organisations.
**Your organisation** is the named place/group where you work

**Your role** is what you do in your organisation (if you occupy multiple roles, please select one role to answer the survey from a single perspective).

**Information for Completion**

The deadline for completion is **December 31st**.

The survey will cover the following key areas:

	Identifying and assessing palliative care needs within your service
	Response to needs within your service
	Details of your service improvement
	About you and where you work

 

Your commitment should be a total of approximately 25 minutes.

 

 

**Navigating the Survey**

 

Each time you click one of the blue arrow buttons at the bottom of each survey page, to move back and forth through the survey, your responses and progress will be saved. A progress bar in the survey will show you what stage of the survey you have reached. You therefore have the option, if you prefer, to complete the survey in multiple sittings. 

 

Please Note  Your saved progress relies on you using the same internet browser and the history of your progress being remembered in your internet browser's 'cookies'. Therefore, if you use a different internet browser, or you clear your browser history or 'cookies', then you will lose your responses and will be unable to continue from where you had previously been in the survey. 

 

Please Note  We recommend, for better usability and navigation through the survey, that where possible you use a laptop or desktop device to complete this survey.

 

If you have any problems with using the survey, then please contact Dr Richard Green by email: [richard.green@surrey.ac.uk](mailto:richard.green@surrey.ac.uk?subject=PALLUP%20Study%20-%20Survey%20Query&amp;body=Dear%20Richard%2C%20%0A%0A)

|  |
| --- |

Consent

 

Please read the [Participant Information Sheet](https://drive.google.com/file/d/1I_zITnHS349xpPIl2DqGOpevgLEA-y-O/view?usp=sharing) prior to participating in this online survey.

 

Your consent to take part in this research will be assumed if you complete a survey. Once a survey response has been submitted, that data cannot be withdrawn. 

 

 

**Please read and then click on to select each of the statements below (this will highlight the statement in blue with white text) to indicate your agreement with the statement:**

(**Please Note:** the first and second statements below must be selected to begin this survey, otherwise you will proceed directly to the end of the survey)

 

- I have read the study information and consent to take part in this study. 

- I understand that my responses will remain confidential and that I can withdraw from the study at any time. I understand that once a questionnaire has been submitted, the data cannot be withdrawn. 

- I agree that my personal contact details be stored by the research team who may wish to invite me to participate in follow-up research for this project or in future studies being conducted at the University of Surrey. I understand that selecting this box does not oblige me to take part in follow-up or future research. 

End of Block: Information and Consent

Start of Block: Identification and Addressing Needs

|  |
| --- |

Does your service address the needs of older people living with advancing frailty at home, who are thought to be in the final years of life? 

**Please Note** if you select 'No' or 'Don't Know / Unsure' for this question then you should not proceed with this survey.

- Yes 

- No 

- Don't Know / Unsure 

|  |
| --- |

Has your service been involved within the last five years in introducing a service improvement change to address the palliative care needs of older people living with advancing frailty at home?

**Please Note** if you select 'No' or 'Don't Know / Unsure' for this question then you should not proceed with this survey.

- Yes 

- No 

- Don't Know / Unsure 

|  |
| --- |

In what way has your service introduced service improvement change(s)?

(Please select all that apply)

- Service improvement that has changed how care is provided within your existing service 

- Service improvement that has changed how care is provided by working with a new organisation(s) and/or Health/Social/Voluntary care worker outside your existing service 

- Don't Know / Unsure 

**The following questions ask you to consider the components of the service pathway for an older person with advancing frailty** 

**Please note:** Not all questions will apply to your service, please respond only to those that are relevant.

**Identification**

How do people access your service? (Please select all that apply)

- Self-referral 

- Direct referral 

- Other (please specify) __________________________________________________

|  |
| --- |

Do you identify frailty in your service?

- Yes 

- No 

- Don't Know / Unsure 

|  |
| --- |

How is frailty identified? (Please select all that apply)

- Clinical discussion(s) with the Multidisciplinary Team 

- Clinical discussion(s) with the Multidisciplinary Team **and** Family / Older Person 

- Timed-up-and-go test ( 

- Grip strength 

- PRISMA 7 questionnaire 

- Clinical frailty scale (CFS) 

- Edmonton frail scale 

- Barthel Scale/Index (BI) 

- Population-level tool e.g. the electronic Frailty Index (eFI) 

- Other population management or individual identification tools (please describe) __________________________________________________

- Don't Know / Unsure 

|  |
| --- |

Do you identify those thought to be nearing the end of life in your service?

- Yes 

- No 

- Don't Know / Unsure 

|  |
| --- |

How are those thought to be nearing the end of life identified? (Please select all that apply)

- Clinical discussion(s) with the Multidisciplinary Team 

- Clinical discussion(s) with the Multidisciplinary Team **and** Family / Older Person 

- The Gold Standards Framework Proactive Identification Guidance (PIG) 

- The Surprise Question 

- The Supportive and Palliative Care Indicators Tool [SPICT] 

- Planned end of life meeting(s) 

- Identifying awareness of increasing end of life care need 

- Other (please describe) __________________________________________________

- Don't Know / Unsure 

As an estimate, what percentage of the older people living with advancing frailty using your organisation's care service over the last twelve months are...? 

(Please slide the scale indicator below for each item to give an estimated percentage, or select the 'Unknown / Cannot Estimate' option if you do not wish to estimate)

|  | Unknown / Cannot Estimate |
| --- | --- |

|  | 0 | 10 | 20 | 30 | 40 | 50 | 60 | 70 | 80 | 90 | 100 |
| --- | --- | --- | --- | --- | --- | --- | --- | --- | --- | --- | --- |

| Men? |  |
| --- | --- |
| From Black and Minority Ethnic Backgrounds? |  |
| From low-income households? |  |
| From single occupancy households? |  |

As an estimate, what percentage of the older people living with advancing frailty in your geographical catchment area do you think your service has seen in the last twelve months?

|  | Unknown / Cannot Estimate |
| --- | --- |

|  | 0 | 10 | 20 | 30 | 40 | 50 | 60 | 70 | 80 | 90 | 100 |
| --- | --- | --- | --- | --- | --- | --- | --- | --- | --- | --- | --- |

| Percentage |  |
| --- | --- |

| Page Break |  |
| --- | --- |

**Reviewing and Addressing Needs**

**The research team recognises existing pressures on services and demands on resources. Honest answers about where needs are not being addressed help us to identify where more support is needed, and the research team are most interested in what is realistically possible for services.**

**Reminder:**

Not all questions will apply to your service, please respond only to those that are relevant.

|  |
| --- |

Thinking about the care that is provided in your service, how often do the following take place...?

|  | Always/Often | Sometimes | Rarely/Never | Will Signpost/ Refer On | Don't Know / Unsure | N/A for my service |
| --- | --- | --- | --- | --- | --- | --- |
| Review the needs of an older person at point of first contact |  |  |  |  |  |  |
| Review the needs of an older person at each subsequent consultation after first contact |  |  |  |  |  |  |
| Review the needs of an older person using a tailored and/or holistic assessment |  |  |  |  |  |  |
| Involve an older person and their family in reviewing their care needs |  |  |  |  |  |  |
| Review the needs of an older person's unpaid Carer |  |  |  |  |  |  |
| Identify what matters most to an older person (their priorities, preferences and wishes) |  |  |  |  |  |  |
| Plan for different possibilities and outcomes for current and future needs in consultation with an older person |  |  |  |  |  |  |
| Have Multidisciplinary Team (MDT) discussions |  |  |  |  |  |  |

|  |
| --- |

Which of the following does your service primarily provide support for...? 
(Please select all that apply)

**Please note**: 'support' can be either addressing a need or signposting/referring on to other services

- Physical health needs 

- Mental health needs 

- Practical daily living needs 

- Spiritual, religious, and / or cultural needs 

- Older people's strengths 

- Older people's coping strategies 

- Other (please describe) __________________________________________________

How does your service review older people's strengths and / or coping strategies?

________________________________________________________________

________________________________________________________________

________________________________________________________________

________________________________________________________________

________________________________________________________________

|  |
| --- |

Thinking about specific physical health needs, does your service provide support for...?

|  | Always/Often | Sometimes | Rarely/Never | Will Signpost/ Refer On | Don't Know / Unsure | N/A for my service |
| --- | --- | --- | --- | --- | --- | --- |
| Pain |  |  |  |  |  |  |
| Mobility |  |  |  |  |  |  |
| Hearing and/or Visual Difficulties |  |  |  |  |  |  |
| Sleep Disturbance |  |  |  |  |  |  |
| Medicines Use |  |  |  |  |  |  |
| Long Term Disease Management |  |  |  |  |  |  |
| Memory Loss |  |  |  |  |  |  |
| Acute Confusion |  |  |  |  |  |  |

|  |
| --- |

Thinking about specific psychological / mental health needs, does your service provide support for...?

|  | Always/Often | Sometimes | Rarely/Never | Will Signpost/ Refer On | Don't Know / Unsure | N/A for my service |
| --- | --- | --- | --- | --- | --- | --- |
| Low Mood |  |  |  |  |  |  |
| Anxiety |  |  |  |  |  |  |
| Managing Loss / Bereavement |  |  |  |  |  |  |
| Loneliness |  |  |  |  |  |  |

|  |
| --- |

Thinking about specific practical needs, does your service provide support for...?

|  | Always/Often | Sometimes | Rarely/Never | Will Signpost/ Refer On | Don't Know / Unsure | N/A for my service |
| --- | --- | --- | --- | --- | --- | --- |
| Staying Active |  |  |  |  |  |  |
| A Programme of Rehabilitative Care to Restore and Improve  Daily Living Abilities |  |  |  |  |  |  |
| Staying Connected with People and Places |  |  |  |  |  |  |
| Relationships with Family |  |  |  |  |  |  |

|  |
| --- |

Thinking about specific spiritual, religious, and / or cultural needs, does your service provide support for...?

|  | Always/Often | Sometimes | Rarely/Never | Will Signpost/ Refer On | Don't Know / Unsure | N/A for my service |
| --- | --- | --- | --- | --- | --- | --- |
| Spiritual, religious and/or cultural beliefs and practices |  |  |  |  |  |  |
| Culturally sensitive care (e.g. interpreters, diet) |  |  |  |  |  |  |
| Recognising and responding to existential questions older people may have in the final years of life |  |  |  |  |  |  |

For your service, how would you prioritise these needs...? 
(**Please select a maximum of 5 relevant needs to your service** and rank them in order of highest to lowest priority)

(To select an item click on it and drag it to the box on the right)

| Priority List of Needs (Highest (=1) at the top down to the lowest) |
| --- |
| ______ Pain |
| ______ Mobility |
| ______ Hearing and/or Visual Difficulties |
| ______ Sleep Disturbance |
| ______ Medicines Use |
| ______ Long Term Disease Management |
| ______ Memory Loss |
| ______ Acute Confusion |
| ______ Low Mood |
| ______ Managing Loss / Bereavement |
| ______ Loneliness |
| ______ Staying Active |
| ______ A Programme of Rehabilitative Care to Restore and Improve  Daily Living Abilities |
| ______ Staying Connected with People and Places |
| ______ Relationships with Family |
| ______ Spiritual, religious and/or cultural beliefs and practices |
| ______ Culturally sensitive care (e.g. interpreters, diet) |
| ______ Recognising and responding to existential questions older people may have in the final years of life |

Which needs (if any) do you wish your service could address but currently does not? (**Please select a maximum of 3 needs**)

(To select an item click on it and drag it to the box on the right) 

| Needs Wishlist (Please select up to 3 needs) |
| --- |
| ______ Pain |
| ______ Mobility |
| ______ Hearing and/or Visual Difficulties |
| ______ Sleep Disturbance |
| ______ Medicines Use |
| ______ Long Term Disease Management |
| ______ Memory Loss |
| ______ Acute Confusion |
| ______ Low Mood |
| ______ Managing Loss / Bereavement |
| ______ Loneliness |
| ______ Staying Active |
| ______ A Programme of Rehabilitative Care to Restore and Improve  Daily Living Abilities |
| ______ Staying Connected with People and Places |
| ______ Relationships with Family |
| ______ Spiritual, religious and/or cultural beliefs and practices |
| ______ Culturally sensitive care (e.g. interpreters, diet) |
| ______ Recognising and responding to existential questions older people may have in the final years of life |

Are there any important needs that your service addresses that have not been identified in the survey? (If so, please describe)

________________________________________________________________

________________________________________________________________

________________________________________________________________

________________________________________________________________

________________________________________________________________

|  |
| --- |

Which of the following tailored and/or holistic assessment methods does your service use? (Please select all that apply)

- Palliative Outcome Scale (POS) 

- Integrated Palliative Outcome Scale (IPOS) 

- Specialist Palliative Care Assessment (e.g. Outcome Assessment and Complexity Collaborative (OACC) suite of measures) 

- Comprehensive Geriatric Assessment (CGA) 

- An assessment template specific to your service (please describe) __________________________________________________

- A personalised assessment focussing on what is important to the older person (e.g. a GP consultation) 

- A patient or carer held tool to monitor symptoms or changes 

- Goals of care to structure older people's consultations 

- Other(s) (please describe) __________________________________________________

|  |
| --- |

Which of the following methods does your service use to support unpaid carers? (Please select all that apply)

- End of life focus care tools (e.g. Carer Support Needs Assessment Tool - CSNAT) 

- A local council carers assessment 

- An assessment template specific to your service (please describe) __________________________________________________

- Other (please describe) __________________________________________________

End of Block: Identification and Addressing Needs

Start of Block: Response

**Service structures to support your service response**

|  |
| --- |

Which of the following service structures does your service provide?

|  | Always/Often | Sometimes | Rarely/Never | Will Signpost/ Refer On | Don't Know/ Unsure | N/A for my service |
| --- | --- | --- | --- | --- | --- | --- |
| 24 hours service access |  |  |  |  |  |  |
| Advance / anticipatory / future care planning conversations |  |  |  |  |  |  |
| Medication management at person's home |  |  |  |  |  |  |
| A shared digital care records system (e.g. the Electronic Palliative Care Co-ordination System) that can be accessed by different professions |  |  |  |  |  |  |
| Consent and patient support to engage with shared digital records |  |  |  |  |  |  |
| Remote telehealth services (e.g. video consultations) |  |  |  |  |  |  |
| Assign a named person or group for ongoing contact |  |  |  |  |  |  |
| Assign a care coordinator to facilitate communication between different professionals and services |  |  |  |  |  |  |
| Access to mobility equipment and/or household modifications |  |  |  |  |  |  |
| Connections with local / national voluntary organisations |  |  |  |  |  |  |
| Support to community and public health initiatives promoting health and wellbeing and living and dying well |  |  |  |  |  |  |

How does your organisation provide 24 hours service access?

- Having an organisational single point of contact that is always available 

- Providing some cover and then signposting to other services at other times 

|  |
| --- |

How does your service provide medications management at home? 
(Please select all that apply)

- Specialist palliative care medications 

- Arranging just-in-case medications in home care settings 

- Working with community pharmacists 

- Review of medicines use 

- Other (please describe) __________________________________________________

|  |
| --- |

Which technology-facilitated communication methods does your service use for the facilitation and delivery of care remotely? (Please select all that apply)

- Telephone 

- Video Calls 

- Text Services 

- Email 

- Remote monitoring technologies for health conditions 

- Smartphone Apps 

- Other (please describe) __________________________________________________

|  |
| --- |

How does your service provide advance / anticipatory / future care planning? 
(Please select all that apply)

- Conversations over time 

- Conversations with others (e.g. surrogate decision-makers) 

- Providing information about Power of Attorney 

- Support with planning for future care decisions and preferences (e.g. completing Do Not Attempt Resuscitation forms, Recommended Summary Plan for Emergency Care and Treatment (ReSPECT)) 

- Support with managing practical affairs associated with dying (e.g will, finances, funeral arrangement) 

- Consent and information regarding shared Digital Records 

- Other (please describe) __________________________________________________

To what shared digital care records system(s) does your service have access?

________________________________________________________________

________________________________________________________________

________________________________________________________________

________________________________________________________________

________________________________________________________________

|  |
| --- |

Do older people and their family have access to this shared digital care records system?

- Yes 

- No 

What (if any) barriers and/or challenges exist for any of your service providers in accessing this shared digital care records system?

________________________________________________________________

________________________________________________________________

________________________________________________________________

________________________________________________________________

________________________________________________________________

|  |
| --- |

Which professions or specialist groups will you signpost/refer people to...? 
(Please select all that apply)

|  | Always/Often | Sometimes | Rarely/Never | Unavailable | N/A for my service |
| --- | --- | --- | --- | --- | --- |
| Older People / Frailty |  |  |  |  |  |
| Community Nurse |  |  |  |  |  |
| Mental Health |  |  |  |  |  |
| Direct Social Care (e.g. Domiciliary Care) |  |  |  |  |  |
| Occupational Therapy |  |  |  |  |  |
| Physiotherapy |  |  |  |  |  |
| Dietician |  |  |  |  |  |
| Community Pharmacy |  |  |  |  |  |
| Paramedic |  |  |  |  |  |
| Voluntary Worker |  |  |  |  |  |
| Faith Worker |  |  |  |  |  |
| Social Worker |  |  |  |  |  |
| Chiropodist / Podiatrist |  |  |  |  |  |
| Specialist Palliative Care |  |  |  |  |  |
| Social Prescriber |  |  |  |  |  |
| Speech Therapist |  |  |  |  |  |
| Dental Care |  |  |  |  |  |
| Other |  |  |  |  |  |

|  |
| --- |

Does your service engage with other providers to identify those who might benefit from your service?

- Yes 

- No 

|  |
| --- |

How does your service engage with other providers to identify those who might benefit from your service? (Please select all that apply)

- Structured times to meet and discuss possible referrals  

- Specific outreach role in other organisations 

- Staff co-located with other services 

- Literature available on website(s) and paper literature 

- Known to local end of life and older people's groups 

- Other (please describe) __________________________________________________

|  |
| --- |

Does your service promote itself to the general public?

- Yes 

- No 

|  |
| --- |

How does your service promote itself to the general public (select all that apply)

- Service Website / Webpage 

- Email Subscription Mailing List 

- Social Media (e.g. Facebook, Twitter) 

- Hardcopy Literature 

- Newspaper advertisements 

- In-reach events (welcoming the public into a service) 

- Outreach events (providing information in community spaces about the service) 

- Other (please describe) __________________________________________________

End of Block: Response

Start of Block: System Support and Service Improvement

**The following questions are about the systems in place within your service to support how you work**

|  |
| --- |

**Does your service / organisation provide education and training for...?**

|  | Yes | No | Don't Know | N/A for my service |
| --- | --- | --- | --- | --- |
| Your staff to promote confidence and capability in end-of-life care for older people with advancing frailty |  |  |  |  |
| Other services to promote confidence and capability in end-of-life care for older people with advancing frailty |  |  |  |  |
| Communication that considers any sensory needs an older person may have (e.g. hearing or sight difficulties) |  |  |  |  |
| Communication with other care providers effectively to manage transitions in care |  |  |  |  |
| On safeguarding for vulnerable people |  |  |  |  |

|  |
| --- |

**Does your service / organisation provide...?**

|  | Yes | No | Don't Know | N/A for my service |
| --- | --- | --- | --- | --- |
| Outreach to support/educate unpaid carers of older people |  |  |  |  |
| Outreach to other care sectors |  |  |  |  |
| Support services for the wellbeing of staff/volunteers |  |  |  |  |
| Appropriate information and education available for patients and families in accessible formats for them |  |  |  |  |

**The following questions are about service improvement changes that have taken place within your service**

|  |
| --- |

Does your service have systems that support collecting data and estimating service costs?

- Yes 

- No 

- Don't Know / Unsure 

If you have any information on the cost of your service that you would be willing to share, then please write this below.

________________________________________________________________

________________________________________________________________

________________________________________________________________

________________________________________________________________

________________________________________________________________

|  |
| --- |

Has your service undertaken any evidence building (e.g. reviews, evaluations) about your service within the last five years?

- Yes 

- No 

- Don't Know / Unsure 

What evidence building has been undertaken?

________________________________________________________________

________________________________________________________________

________________________________________________________________

________________________________________________________________

________________________________________________________________

How has your service changed in response to the Covid-19 pandemic?

________________________________________________________________

________________________________________________________________

________________________________________________________________

________________________________________________________________

________________________________________________________________

What is the most important service improvement change your service has made in the last five years, why was it undertaken, and why was it the most important?

________________________________________________________________

________________________________________________________________

________________________________________________________________

________________________________________________________________

________________________________________________________________

What has been the impact of this service improvement?

________________________________________________________________

________________________________________________________________

________________________________________________________________

________________________________________________________________

________________________________________________________________

What of the service development do you plan to **keep** and and what do you plan to **change**?

________________________________________________________________

________________________________________________________________

________________________________________________________________

________________________________________________________________

________________________________________________________________

|  |
| --- |

Do you have any plans to scale up your service improvement, to take it to a larger group of people?

- Yes 

- No 

- Don't Know / Unsure 

|  |
| --- |

Do you have any plans to spread your service improvement to other teams locally or in your region?

- Yes 

- No 

- Don't Know / Unsure 

If you have anything further to add to your responses to this survey please do so here.

________________________________________________________________

________________________________________________________________

________________________________________________________________

________________________________________________________________

________________________________________________________________

End of Block: System Support and Service Improvement

Start of Block: About You and Where You Work

**About You and Where You Work**

 
Lastly, please complete the following questions that will help us understand more about your survey responses and record study recruitment accurately, to inform future research funding.

Your responses will remain anonymous and no identifiable information will be presented in research outputs.

What is the name of the service / organisation that you work for in your primary role?

(This allows us to record your recruitment in our study, which informs future research funding allocation for the National Institute of Health Research, our study's funder - you are not required to answer this question if you do not wish)

________________________________________________________________

What is the post code for this service / organisation? 

(This allows us to record your recruitment in our study, which informs future research funding allocation for the National Institute of Health Research, our study's funder - you are not required to answer this question if you do not wish)

________________________________________________________________

If your organisation has a specific named service within it, or your organisation is part of a service comprised of multiple organisations, to which you belong in your primary role, then please provide the name of this service below.

________________________________________________________________

What is your primary role?

- Service/Patient-facing (either clinical or non-clinical) 

- Organisational Leadership or Governance 

What is your grade?

- Team Member 

- Team Leader or Supervisor 

- Deputy Manager or Team Manager 

- Specialist Coordinator (Leading in a specialist area of care, e.g. dementia) 

- Manager 

- Senior Management / Executive 

- Other (please describe) __________________________________________________

Within which sector is your primary role?

- Health Care 

- Social Care 

- Voluntary Care 

- Other (please describe) __________________________________________________

|  |
| --- |

Within which setting is your primary role?

- Acute 

- Primary 

- Secondary 

- Intermediate 

- Community 

- Other (please describe) __________________________________________________

|  |
| --- |

Within which professional group(s) do you work? (Please select all that apply)

- Older People / Frailty 

- Community Nurse 

- Mental Health 

- Direct Social Care (e.g. Domiciliary Care) 

- Occupational Therapy 

- Physiotherapy 

- Dietician 

- Community Pharmacy 

- Paramedic 

- Voluntary Worker 

- Faith Worker 

- Social Worker 

- Chiropodist / Podiatrist 

- Specialist Palliative Care 

- Social Prescriber 

- Speech Therapist 

- Dental Care 

- Care Commissioner 

- Non-Clinical Leadership or Governance 

- Other (please describe) __________________________________________________

|  |
| --- |

In which English region(s) is your organisation located? (Please select all that apply)

- East Midlands 

- East of England 

- Greater London 

- North East 

- North West 

- South East 

- South West 

- West Midlands 

- Yorkshire and the Humber 

- Other Area - Please Specify __________________________________________________

- Don't Know / Unsure 

|  |
| --- |

What is your ethnic group? 

- Asian or Asian British 

- Black or Black British 

- Mixed 

- White 

- Other (Please Specify) __________________________________________________

- Prefer not to say 

|  |
| --- |

What is your gender identity? 

- Male 

- Female 

- Non-binary / Prefer to self-describe 

- Prefer not to say 

|  |
| --- |

What is your first name? 

________________________________________________________________

|  |
| --- |

What is your surname?

________________________________________________________________

|  |
| --- |

What is your email address?

________________________________________________________________

End of Block: About You and Where You Work
